# Patients’ and family caregivers’ experiences and perceptions about factors hampering or facilitating patient empowerment for self-management of hypertension and diabetes in Cameroon

**DOI:** 10.1186/s12913-022-08750-4

**Published:** 2022-11-21

**Authors:** Amélie Mogueo, Barthelemy Kuate Defo

**Affiliations:** 1 Programme en Population, Nutrition et Une-Santé Transnationales, Infranationales, Nationales et Continentales (PRONUSTIC) / Program in Transnational, Subnational, National and Continental Population, Nutrition and One-Health (PRONUSTIC), University of Montreal, Montreal, Quebec H3T 1N8 Canada; 2 Department of social and preventive medicine, School of public health, University of Montreal, 7101 Avenue du Parc, Montreal, Quebec H3N 1X9 Canada; 3 Department of demography, University of Montreal, Pavillon Lionel-Groulx, C. P. 6128, succursale Centre-ville, Montreal, Quebec H3C 3J7 Canada; 4 Public Health Research Center (CReSP), University of Montreal, 7101 Avenue du Parc, C.P. 6128 Succursale Centre-Ville, Montreal, Quebec H3C 3J7 Canada

**Keywords:** Patient empowerment, Self-management, Noncommunicable diseases, Hypertension, Diabetes, Healthcare delivery, Barriers, Facilitators, Qualitative methods, Africa

## Abstract

**Background:**

Noncommunicable diseases like hypertension and diabetes require long-term management, and are financially draining for patients and their families bearing the treatment costs, especially in settings where the inadequacy or non-existence of the health insurance system prevails. Patient empowerment-focused interventions have been shown to improve adherence to therapeutic regimens and decrease unnecessary health care utilization and costs. This study aims to examine enabling and impeding factors to the development of patient empowerment in a resource-limited setting like Cameroon.

**Methods:**

We used qualitative methods entailing three levels of investigation and involving a public primary healthcare hospital in Yaoundé, Cameroon. Data were collected through 40 semi-structural interviews with patients having hypertension or diabetes and their family caregivers, one focus group discussion with six patients, 29 observations of consultations of patients by specialist physicians, seven observations of care received by inpatients from generalist physicians, and nine documents on the management of hypertension or diabetes. A novel approach combining thematic and lexicometric analyses was used to identify similarities and differences in barriers and facilitators associated with patient empowerment at different levels of the healthcare delivery system in Cameroon.

**Results:**

Barriers generally outnumbered facilitators. There were particularities as well as commonalities in reported facilitators and barriers linked to patient empowerment from different experiences and perspectives of outpatients, inpatients and their family caregivers, given the healthcare services and organization of health personal and resources that deliver healthcare services to meet the health needs of patients with hypertension or diabetes in Cameroon. While specific factors identified by patients were directly related to the self-management of their disease at the individual level, family caregivers were mainly focused on factors present at organizational and central levels, which are indirectly related to the management of the diseases and beyond the control of patients and families.

**Conclusions:**

The preponderance of individual-level factors linked to patient empowerment more than those at the central and hospital/organizational levels calls for due attention to them in the multilevel design and implementation of patient empowerment interventions in resource-limited settings like Cameroon. Accounting for patient’s and families’ perspectives and opinions may be key to improving healthcare delivery.

**Supplementary Information:**

The online version contains supplementary material available at 10.1186/s12913-022-08750-4.

## Introduction

The global disability-adjusted-life-years from noncommunicable diseases (NCDs) startlingly increased by 41.1% between 1990 and 2017 [1]. This increase is driven by the rapidly growing burden of NCDs in low- and middle-income countries (LMICs), especially hypertension and type 2 diabetes which are among the leading causes of morbidity, mortality, and reduced life expectancy worldwide, especially in African countries like Cameroon [2]. This study focuses jointly on hypertension and diabetes because they are known to coexist in the population; their coexistence confers a dramatically increased risk (about 2–4 fold) of cardiovascular disease, end-stage kidney disease, and death, compared to the normotensive and nondiabetic adults [3]. Moreover, the pathogenic relationship between hypertension and diabetes is bidirectional, their prevalence increases with increasing age [4, 5], and adequate control of blood pressure significantly reduces the risk of diabetic macrovascular and microvascular complications [6]. The prevalence of hypertension and diabetes in Cameroon is estimated at 32.1% [7] and 5.8% [8], respectively. Hypertension and diabetes are long-term management diseases financially draining for patients and their families in Africa and Cameroon notably, given the inadequate or non-existence of a health insurance system [9, 10]. Also, the hospital healthcare in Cameroon remains largely an acute care model with perceived staff shortages and ineffective communication. This has resulted in a high prevalence of patients being non-adherent to therapeutic plans. Thus, there is an urgent need to implement cost-effective patient-based interventions that empower patients to control and manage their own disease, following the World Health Organization’s framework on integrated, people-centred health services, which emphasizes the importance of organizing primary health care around comprehensive needs of people, rather than around a singular focus of specific diseases [11].

Patient empowerment (PE) has gained prominence in the healthcare system, emerging as one of the general principles of the World Health Organization’s global action plan for the prevention and control of NCDs 2013–2020 [12]. A PE approach sees the patient as an actor of care, who can participate in decision making process, develop competency, self-manage his condition, and contribute to continuous improvement in the quality of healthcare delivery. Interventions based on PE or self-management in NCDs have been shown to improve a variety of important health outcomes [13]. These include adherence to therapeutic plans and decreased unnecessary health care utilization and costs in every health care system [14–17]. The PE approach requires contextualized innovative disease management strategies. Implementation research shows that interventions based on PE face generally barriers that need to be identified to optimize its use, as perceived by different groups of key stakeholders such as patients and their families, health professionals and policy makers [18–20]. Research on patients and families’ perceptions and experiences about factors facilitating or hampering the development of PE remains scanty in resource-limited settings [16, 17, 21].

This study aimed to pinpoint influential factors associated with the development of PE in the primary healthcare context of Cameroon, often considered as a microcosm of Africa [7]. Exploring these factors systematically may help policymakers and clinicians in identifying and understanding both facilitators and barriers to empowerment of patients and in tailoring interventions to the identified factors. Given the rising multimorbidity of chronic diseases in primary care and its effect on healthcare utilization and costs worldwide, this study will contribute to inform future interventions to remove barriers to and creating a facilitating environment for PE in the healthcare systems in sub-Saharan Africa for NCDs and the management of hypertension and diabetes.

## Methods

### Conceptual framework

**Fig. 1:**
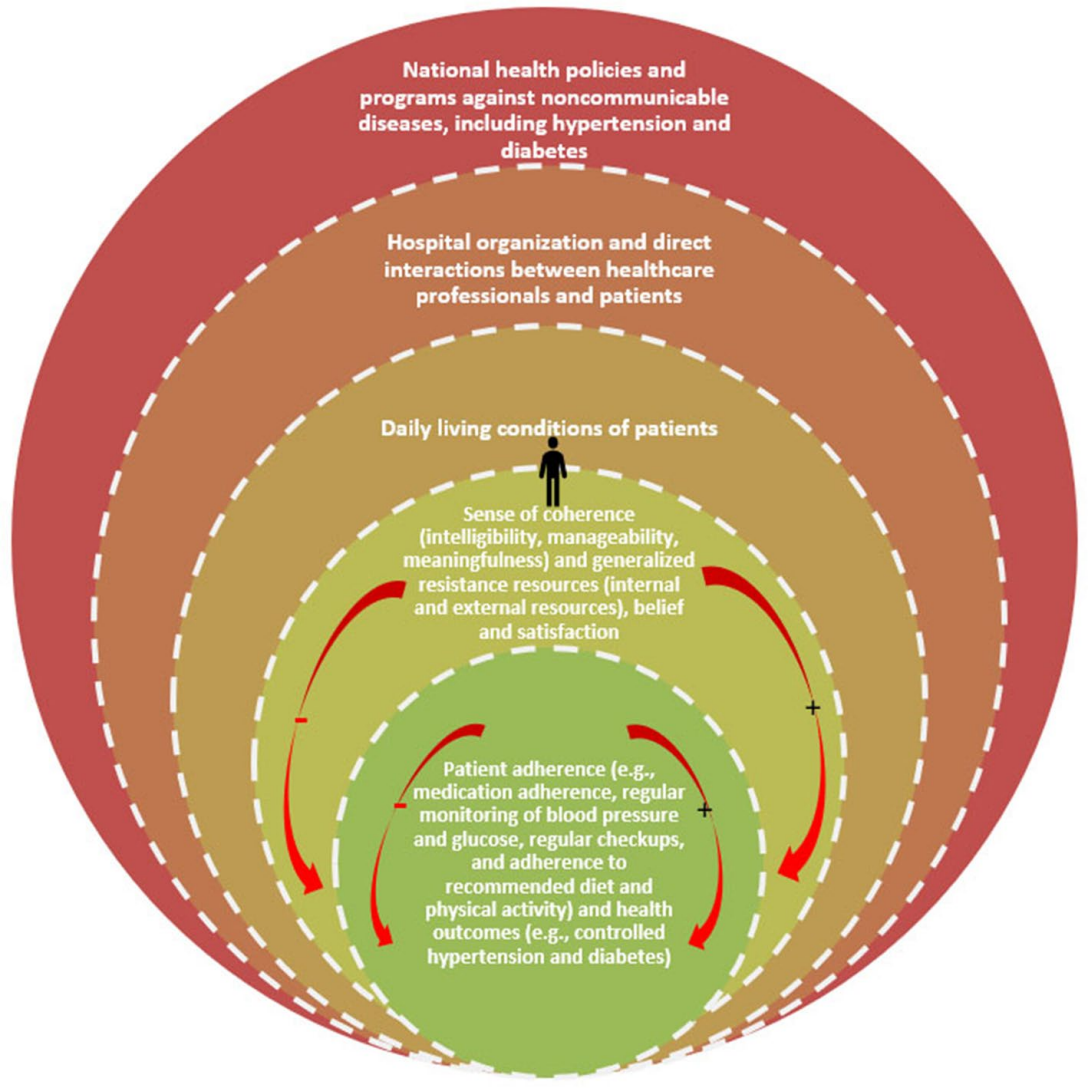
An integrated framework for understanding barriers and facilitators linked to patient empowerment in the management of hypertension or diabetes

Defined as “a process through which people gain greater control over decisions and actions affecting their health” ([22]: 190), PE has been influenced by several theories of health behaviour change [23–25], particularly the salutogenic theory proposed by Antonovsky [26]. We adopted an integrated framework (Fig. 1) to aid data collection, analysis, and presentation of findings; this framework links the salutogenic theory [26, 27] to the health belief model [28, 29] and patient satisfaction theory [30–32].

The salutogenic theory summarizes the PE approach into two main concepts: sense of coherence (SOC) embodied by intelligibility, manageability, meaningfulness; and generalized resistance resources (GRR) embodied by internal and external resources [26]. It suggests that to be empowered and have better health outcome, a patient need to develop a strong intelligibility, manageability, meaningfulness (SOC) with available internal and external resources (GRR). Thus, SOC refers to a patient’s capability to use existing resources (GRR) to understand factors affecting their health (intelligibility), to be able to better manage the diseases (manageability) and to looking at life as worth living (meaningfulness). A stronger SOC is predictive of salutogenesis (or production of health) [27]. The GRR are prerequisites to develop a stronger SOC [26, 27].

The salutogenic theory summarizes the PE approach in two concepts: sense of coherence (SOC) embodied by intelligibility, manageability, meaningfulness; and generalized resistance resources (GRR) represented by resources. To be empowered and have better health outcome, patients need to develop a strong intelligibility, manageability, meaningfulness (SOC) with available internal and external resources (GRR). SOC refers to patients’ capability to use existing resources (GRR) to understand factors affecting their health (intelligibility), to be better able to manage their diseases (manageability) and to look at life as worth living (meaningfulness) [26, 27]. A stronger SOC is predictive of salutogenesis (i.e. production of health). GRR are prerequisites to develop a stronger SOC.

Our framework recognizes that PE axes not only on patient’s SOC and available GRR, but also on patient belief [28, 29] and patient satisfaction [30–32]. Indeed, patients’ beliefs influence their perceptions and experiences of their disease. Personal beliefs influence the understanding of the disease (intelligibility), daily actions taken to manage the disease (manageability), and motivations behind each choice (meaningfulness). Patients’ satisfaction as a positive evaluation of healthcare services means that the more patients are satisfied, the more they will adhere to treatment and hence develop the ability to self-manage their disease and vice-versa (manageability). Patients’ satisfaction also influences their motivation to self-manage their diseases and to make sense of the process (meaningfulness). Therefore, patients’ beliefs and satisfaction influence the SOC (intelligibility, manageability, meaningfulness), and its integration with existing resources (GRR) used by patients. Thus, improving the different components of health behavioral change as depicted in our framework may improve patients’ adherence to treatment and ultimately improve health outcomes. Improved health outcomes may in turn be a source of motivation for people to maintain change, which may reduce the utilization of health care services.

### Study design and setting

We use qualitative methods within a 3-level healthcare system: patient-level, hospital-level, and central/Ministry-level. Patients were recruited in Yaounde (Cameroon) at a primary healthcare district hospital (PHCDH). The PHCH at the peripheral level within the health system in Cameroon is where patient healthcare is operationalized, in line with the health district framework proposed by the World Health Organization [33]. This PHCDH is the first reference for 11 health centres and patients’ gateway to the healthcare system. It included the internal medicine unit having a team of six nurses, two general practitioners (GP), and four specialist physicians (SP), for patients suffering from hypertension/diabetes.

### Participants

We recruited patients with hypertension/diabetes who met our inclusion criteria, and their family caregiver (FC). Eligibility criteria were: had hypertension and/or diabetes for at least 12 months, be hospitalized or followed-up at the selected PHCDH for at least 12 months, and be aged 30 years or older. Pregnant women were excluded. Enrolled patients facilitated access to FC. Hypertension was defined as systolic blood pressure /diastolic blood pressure ≥ 140/90 mmHg. Type 2 diabetes was defined as high levels of blood glucose (Glycated hemoglobin (A1C) ≥ 6.5% or fasting plasma glucose (FPG) ≥ 126 mg/dl or oral glucose tolerance test (OGTT) ≥ 200 mg/dl).

### Data collection

Prior to data collection, we piloted and refined survey instruments based on two 40-minutes interviews with patients and clinic observations. Interviews were recorded and the two authors independently listened to recorded data, checked transcripts for validation, and adjusted interview procedures to better fit our study context. For example, we found that participants’ knowledge about patient empowerment was deficient and decided to explain it.

Data were collected from 07 January to 29 March 2019, through 40 semi-structural interviews, one focus group discussion (FGD), 29 observations of consultations of patients by SP, seven observations of care received by hospitalized patients from GP, nine documents, and field notes. Each interview was conducted at a calm space in the hospital away from other people. One author conducted all interviews using interview guides (Additional file 1), and deliberated with the other author when significant decisions had to be made. The FGD involved six patients previously interviewed individually who agreed to participate. Interviews lasted 20 to 50 minutes and the FGD took 60 minutes. Interviews and FGD were audiotaped and transcribed by one author. The other author independently read all transcripts with corresponding audiotapes, and found no discrepancies.

Direct and passive observations, for 10 to 30 minutes, of consultations of patients took place in SP offices and patients’ hospitalization rooms. We observed interactions between participants and healthcare professionals and took detailed notes. Documents reviewed were health education (*n* = 3), healthcare services for patients (*n* = 2), and national policies/programs on hypertension/diabetes management (*n* = 4).

### Data analysis

All transcripts and field notes were imported into the software package Qualitative Data Analysis (QDA) Miner [34]. Interview data were thematically analyzed by the two authors. We used an initial coding list based on our integrated framework, allowing themes to emerge [35]; to check for consistency, data sources were triangulated by seeking different data sources in the study, and by crosschecking different points of view of stakeholders [36]. Findings from different sources and methods used led to concordant findings.

Over the past four decades or so, computational programs developed for qualitative research data analysis have continued to increase quantitatively and qualitatively for researchers using qualitative research methods. IRAMUTEQ (*Interface de R pour les Analyses Multidimensionnelles de Textes et de Questionnaires* [R Interface for Multidimensional Analysis of Texts and Questionnaires]) is a freely accessible program with complete dictionaries in several languages [37]. It helps organize and separate information, locate text segments and facilitate the codification process in a more objective and systematic fashion than when such process is performed manually. IRAMUTEQ allows different forms of statistical analysis and graphical displays of texts produced from interviews and documents, classic textual analyses, analyses of specificities, similarity analysis and a word cloud, among its features [37, 38]. The IRaMuTeQ software was used to perform the lexicometric analysis [37], including a word cloud and similarity analysis [38]. The word cloud analysis displays the lexicon of words associated with the corpus in the form of a graph where the size of the words is proportional to their frequency. The similarity analysis allows a co-occurrence analysis presented in the form of graphs of associated words. The words are the vertices of the graph, and the links represent the co-occurrences between these words. So, the lexicometric analysis compresses information contained in large texts and provides new perspectives to analyze them.

## Results

### Characteristics of participants

The study involved 40 participants (Tables 1 and 2), with 23 patients, largely represented by women and elderly people, all with either hypertension only, type 2 diabetes only or both hypertension and type 2 diabetes. More than half of them were outpatients who have been living with their disease(s) for 5 years or more. The 17 family caregivers were largely represented by women and people under the age of 60, all caring for patients having hypertension only, type 2 diabetes only or both hypertension and type 2 diabetes (Table 1). Some FC were patients’ wives, while no patient’s husband was FC despite more than half of patients being currently married (Table 2).

**Table 1:** Patient’s disease profile, demographic characteristics, and relationship with the family caregiver

Characteristics		Participants: n (%)		
		Patients	Family caregivers	Total
Overall		23 (57.5)	17 (42.5)	40 (100.0)
Inpatient	Yes	5 (21.7)	5 (29.4)	10 (25.0)
	No	18 (78.3)	12 (70.6)	30 (75.0)
Disease	Hypertension only	8 (34.8)	4 (23.5)	12 (30.0)
	Diabetes only	6 (26.1)	6 (35.3)	12 (30.0)
	Hypertension and diabetes	9 (39.1)	7 (41.2)	16 (40.0)
Patient’s number of years with the disease	< 5 (1–4)	10 (43.5)	–	10 (43.5)
	≥5 (5–30)	13 (56.5)	–	13 (56.5)
Caregiver’s years of caring for the patient	< 5 (1–4)	–	11 (64.7)	11 (64.7)
	≥5 (5–10)	–	6 (35.3)	6 (35.3)
Sex	Female	17 (74.0)	12 (70.6)	29 (72.5)
	Male	6 (26.0)	5 (29.4)	11 (27.5)
Age	< 30 (22–30)	–	3 (17.6)	3 (7.5)
	≥30 (30–64)	–	14 (82.4)	14 (35.0)
	< 60 (46–59)	7 (30.4)	–	7 (17.5)
	≥60 (60–86)	16 (69.6)	–	16 (40.0)
Caregiver’s relationship with the patient	Daughter, n (%)	–	9 (52.9)	9 (52.9)
	Son, n (%)	–	4 (23.5)	4 (23.5)
	Wife, n (%)	–	3 (17.7)	3 (17.7)
	Grandson, n (%)	–	1 (5.9)	1 (5.9)

**Table 2:** Socioeconomic characteristics of patients and family caregivers

Characteristics	Participants: n (%)			
	Patients		Family caregivers	Total
Marital status	Married	14 (60.9)	10 (58.8)	24 (60.0)
	Single	–	7 (41.2)	7 (17.5)
	Divorced or Widowed	9 (39.1)	–	9 (22.5)
Education	None	12 (52.2)	2 (11.8)	14 (35.0)
	Elementary	6 (26.1)	2 (11.8)	8 (20.0)
	Secondary or University	5 (21.7)	13 (76.4)	18 (45.0)
Profession	Housewife	8	2	10
	Trader/businessperson	5	2	7
	Teacher	2	2	4
	Farmer	2	–	2
	University student	–	5	5
	Pastry chef	1	–	1
	Dressmaker	–	2	2
	Healthcare-related profession	1	2	3
	Manager	–	1	1
	Carpenter	1	–	1
	Artist	–	1	1
	Civil servant	1	–	1
	Human Resource Director	1	–	1
	Retired	8	1	9
	Secretary	1	–	1

### Patients’ and family caregivers’ experiences and perspectives

There were 53 barriers (Additional file 2): 4 at the central level: intelligibility (2), manageability (1), external resources (1); 19 at the hospital/organizational level: intelligibility (3), manageability (4), meaningfulness (1), external resources (9), satisfaction (2); 30 at the individual/patient/FC level: intelligibility (5), manageability (5), meaningfulness (4), internal resources (2), external resources (2), adherence and health outcomes (7), belief (5).

We identified 41 facilitators (Additional file 3): 2 at the central level: intelligibility (1) and manageability (1); 12 at the hospital/organizational level: intelligibility (3), manageability (2), internal resources (1), external resources (2), adherence and health outcomes (1), satisfaction (3); 27 at the individual/patient/FC level: intelligibility (7), manageability (4), meaningfulness (5), internal resources (3), external resources (5), adherence and health outcomes (1), belief (1), satisfaction (1).

### Intelligibility

**Fig. 2:**
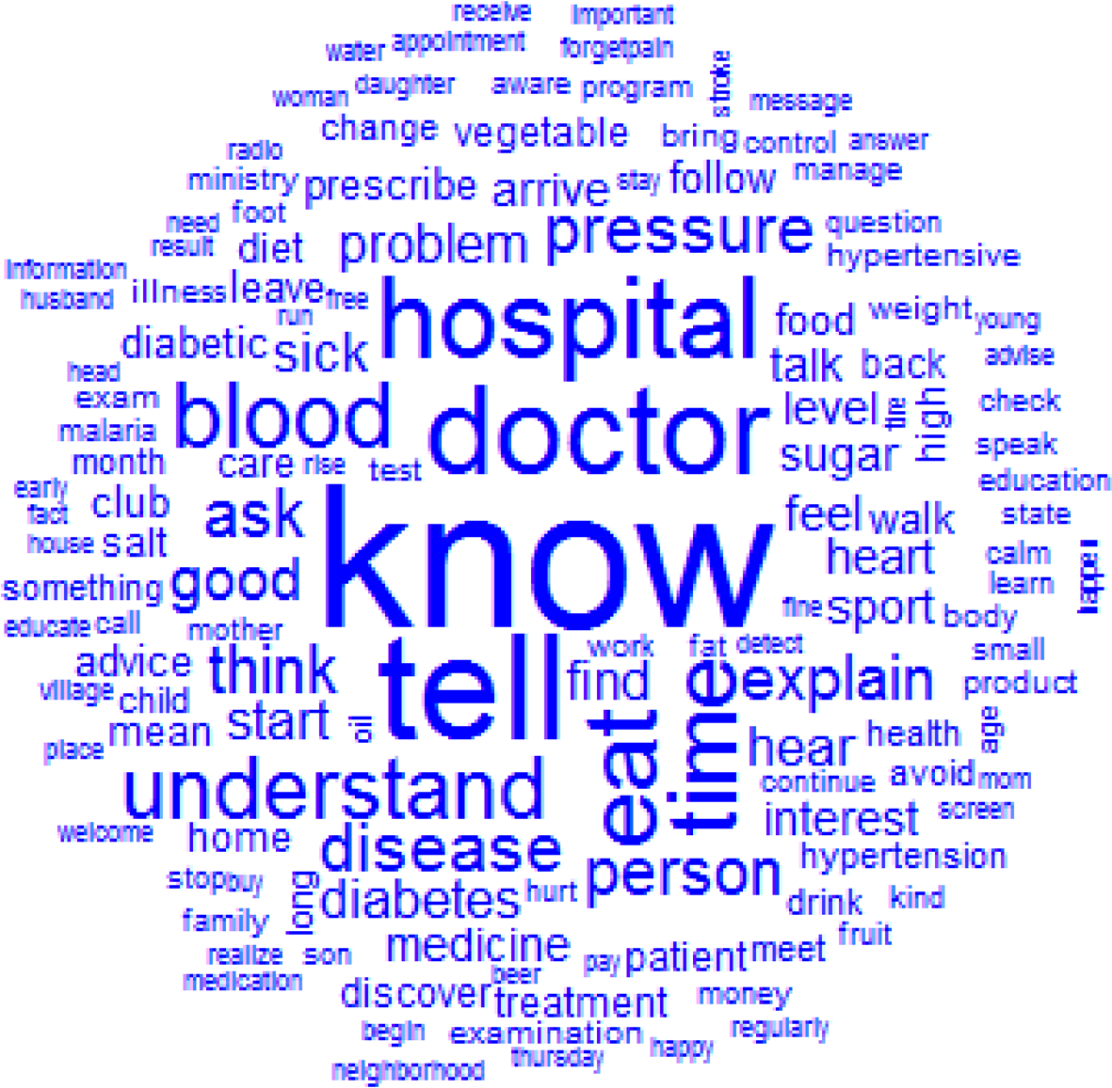
Words used to describe the intelligibility with their frequencies correlating to their size

**Fig. 3:**
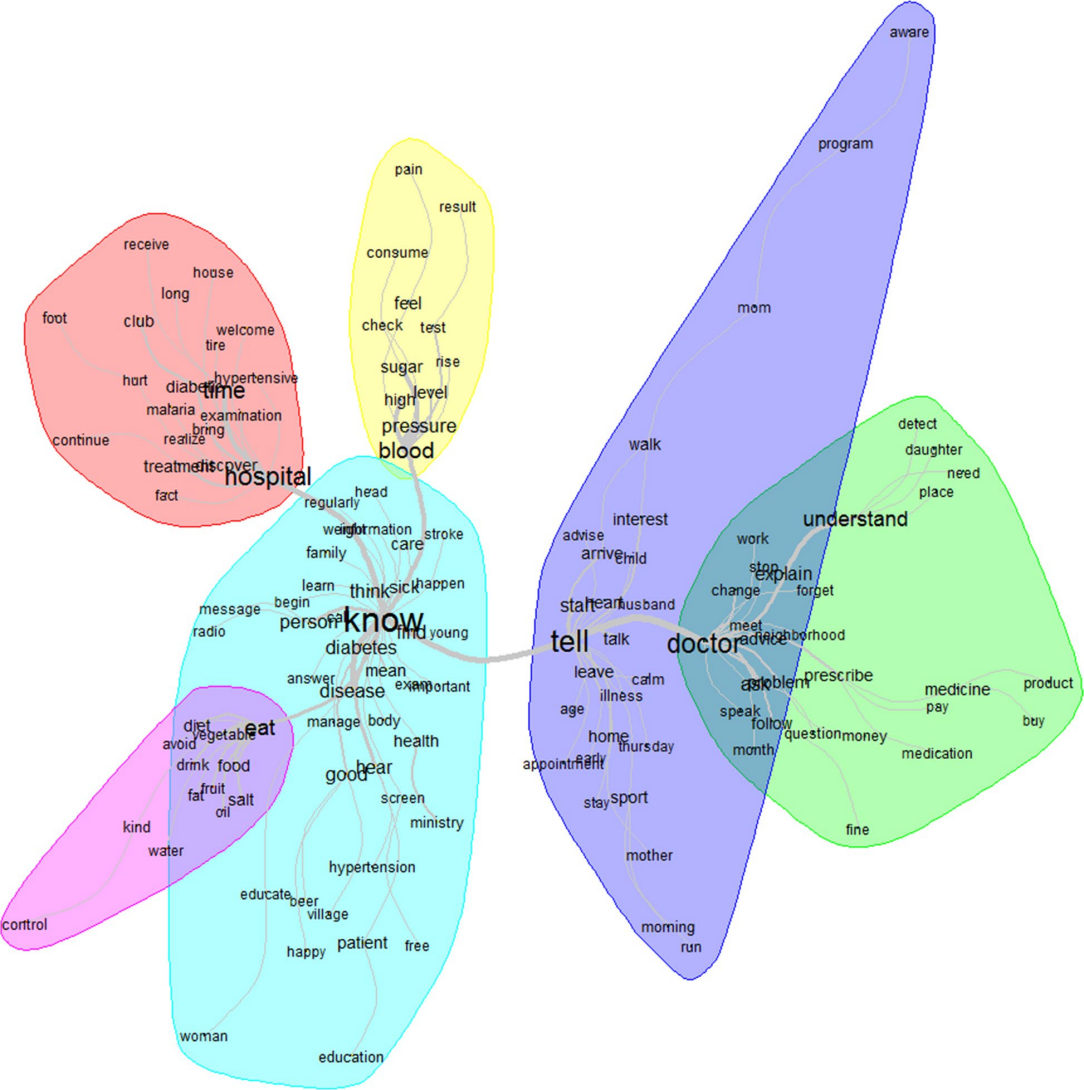
Similarities and links between the different words used to describe the intelligibility

The top 12 words used to describe intelligibility were: know, tell, doctor, hospital, eat, time, blood, understand, disease, pressure, think and person (Fig. 2). Patients’ and family caregivers’ knowledge or information seeking were mainly related to the doctor, hospital, lifestyle (diet/eat), disease control (blood sugar/pressure); the health information access was possible mainly through communication (tell) with the healthcare provider (doctor) (Fig. 3).

Counselling and screening campaigns on NCDs organized by the ministry of health were viewed as limited and not supported by concrete actions helpful to patients.

"… The government needs to talk more about it in neighborhood churches and meetings... They have to send the text messages in our phone...We don’t need the advice only when we come to the hospital.” (FC2)

The *patient education and counseling* (PEC) *for patients suffering from diabetes or both diabetes and hypertension* and consultations with SP were the main channels for accessing information. The PEC was inaccessible to patients with hypertension and their FCs.

“No, I'm not aware of that… I haven’t been told about it since I’ve been here … every time when we arrive, we see the doctor and we left…” (P5)

Some FCs reported that physicians did not explain well the treatment plan, which sometimes caused confusion and discomfort to patients and caregivers. The most educated patients were more active in seeking information about the disease. Yet, the information-seeking process generally began once the disease was diagnosed. Older and less educated patients knew little about these practices, and thought it was the physician’s responsibility. As most of their FCs were younger and more educated, they helped them communicate with physicians to properly identify their needs and propose a suitable treatment plan.

### Manageability

**Fig. 4:**
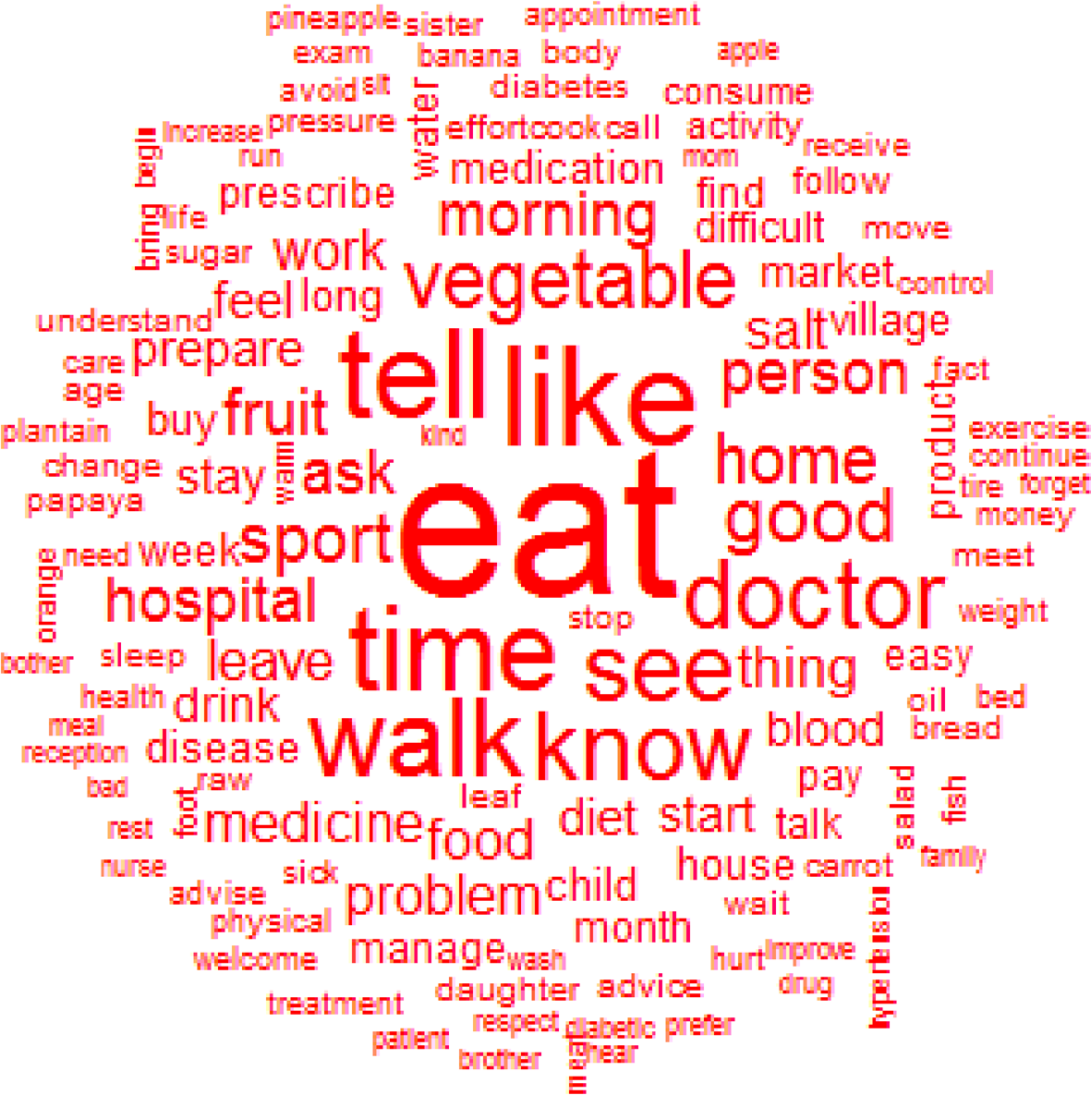
Words used to describe the manageability with their frequencies correlating to their size

**Fig. 5:**
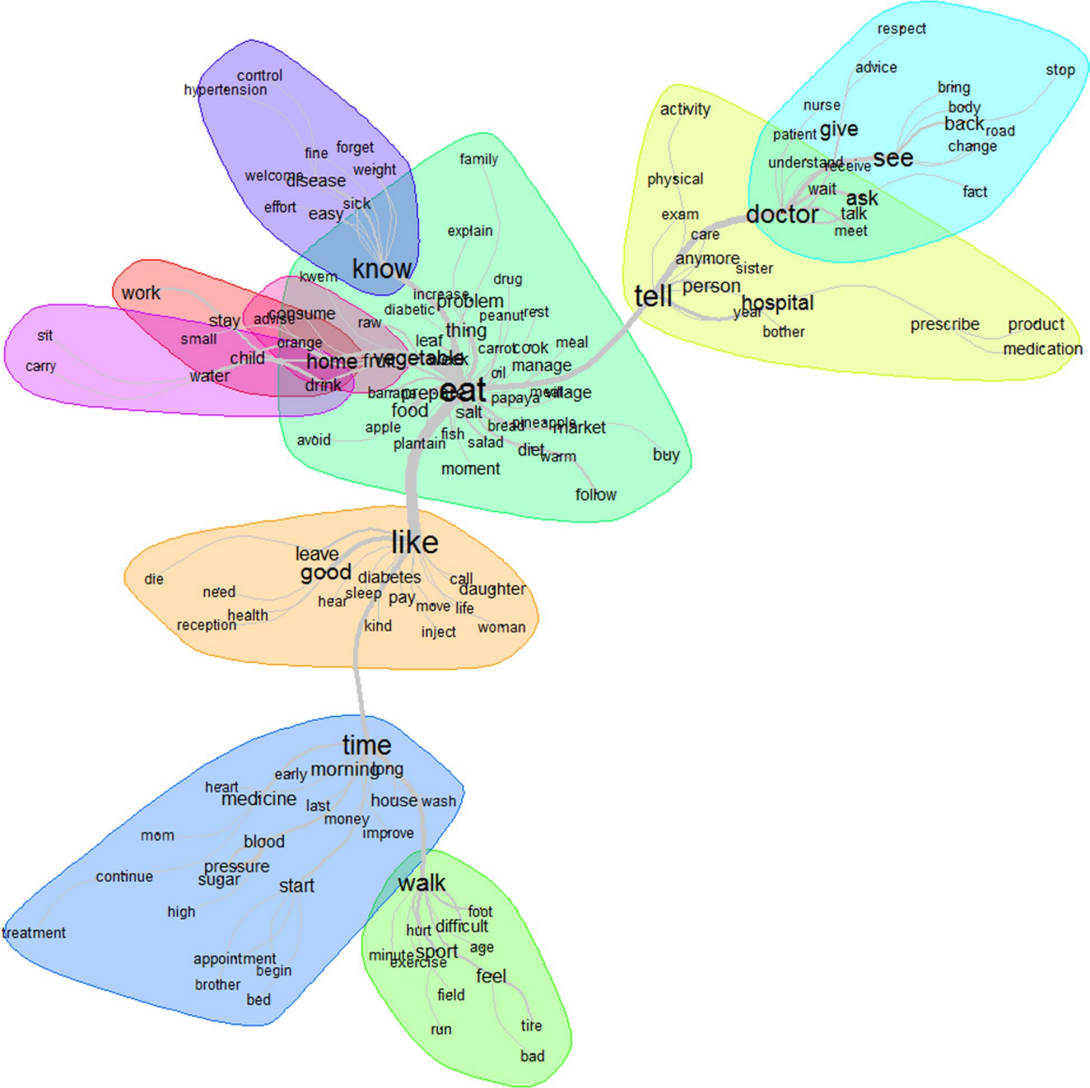
Similarities and links between the different words used to describe the manageability

The top 12 words used to describe manageability were: eat, like, tell, time, walk, know, see, doctor, vegetable, good, sport and home (Fig. 4). The healthy lifestyle, especially the diet (eat) and physical activity (walk), was the most challenging thing for patients and family caregivers as it was associated with comfort/discomfort (like), understanding/misunderstanding (know), and required times and money to have access to a variety of healthy foods (vegetables). The relationship with the healthcare provider (doctor) was also pointed out mainly through the communication (tell) (Fig. 5).

Waiting time was a major barrier highlighted chiefly by FCs of patients with diabetes or both diseases, many of whom had hypoglycemia after several hours of waiting. The quality of reception was also identified as a barrier.

“… She told me today that based only on the quality of the reception, before she sees the doctor, she would have already left the hospital…” (FC13)

The lack of control by the hospital management often led to illegal transactions of medicines, a major barrier for patient empowerment.

“… We don't want nurses to tell you to buy the drugs they have. They tell you, come I have drugs you want while you want to go to a pharmacy… So, we don’t want corruption here…. It’s not good like that.” (FC14)

Being follow-up by SPs was a facilitator of patient empowerment. The trusted patient-physician interaction facilitated adherence to recommended lifestyle and treatment.

“This Doctor has been my doctor for 5 years… he listens, and he gives advices… he explains very well…” (P2)

Sedentary lifestyle was a barrier identified by patients. Self-management of the disease was hampered by stressful financial and familial conditions knotted with out-of-pocket medical expenditures for patients with hypertension. For patients with diabetes, changing eating habits to healthy diet was an important barrier. For patients with both diseases, late diagnosis hindered self-management of their diseases. Women were more actively involved in self-managing their disease than men.

"I asked my children to set up an alarm…to take the medication. That's how I do it in the morning, I get up…, I take my medicine. At noon, I take my medicine… When I hear the ring, I know this is the time to take my medicine…” (P19)

### Meaningfulness

**Fig. 6:**
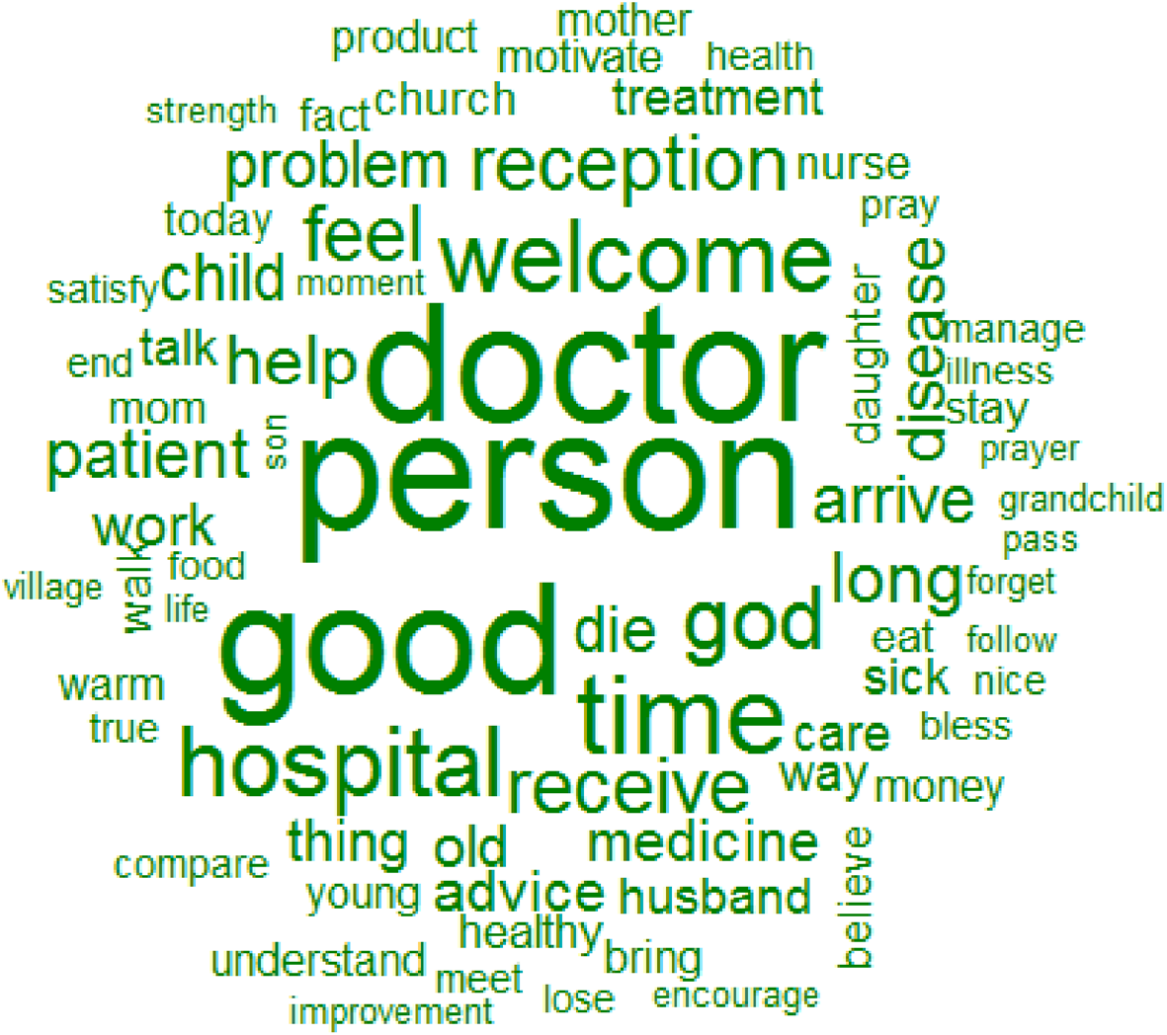
Words used to describe the meaningfulness with their frequencies correlating to their size

**Fig. 7:**
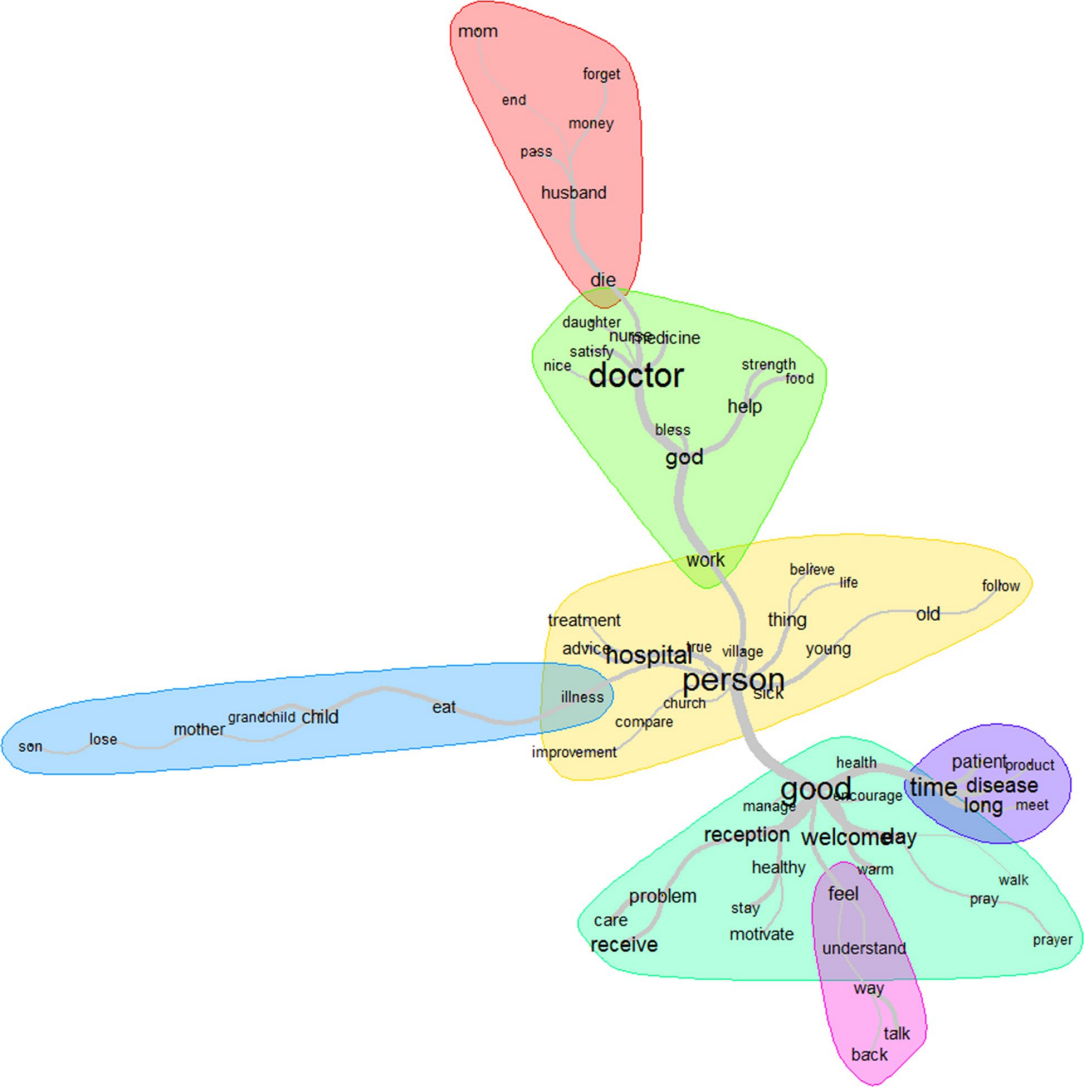
Similarities and links between the different words used to describe the meaningfulness

The top 12 words used to describe meaningfulness were: person, doctor, good, time, welcome, hospital, God, reception, receive, feel, long and help (Fig. 6). The patients’ motivation to be empowered so as to follow the treatment plan was mainly related to the family or neighborhood (person), the reception (good) and the relationship of trust with the healthcare provider (doctor) and the personal belief of patient (God) (Fig. 7).

Some healthcare professionals instilled more fear than confidence in patients by focusing more on the consequences of their disease, a barrier for patients who felt powerless against their disease. Trusted relationships between healthcare professionals and patients reassured the latter that their health will improve.

“… I really believe in the healing process … Me I saw a great improvement, as I came last week Thursday for my result. I went to apply to really see how it works …. the thing (*BS*) drops down from 2.41 to 1.15.” (P21)

Patients’ motivation to manage their diseases was negatively impacted by their perspective that there was no solution against diabetes, especially after a relative’s death like interviewed widows living with these diseases or whose husbands died of hypertension, diabetes, or their complications.

“… I just lost my husband…and my blood sugar and blood pressure have started to rise again… I have no reason to keep fighting for life.” (P1)

Being positive helped some patients develop empowerment to improve their health outcomes. One FC recounted:

"She is quite positive as mom. She wants to live for herself, for her children, her grandchildren, and her great-grandchildren. I think that's what gives her strength … She is a very positive mom even sometimes when we find that she is tired and must rest, she answers no no no you want me to give up? I have to fight …" (FC4)

### Resources

**Fig. 8:**
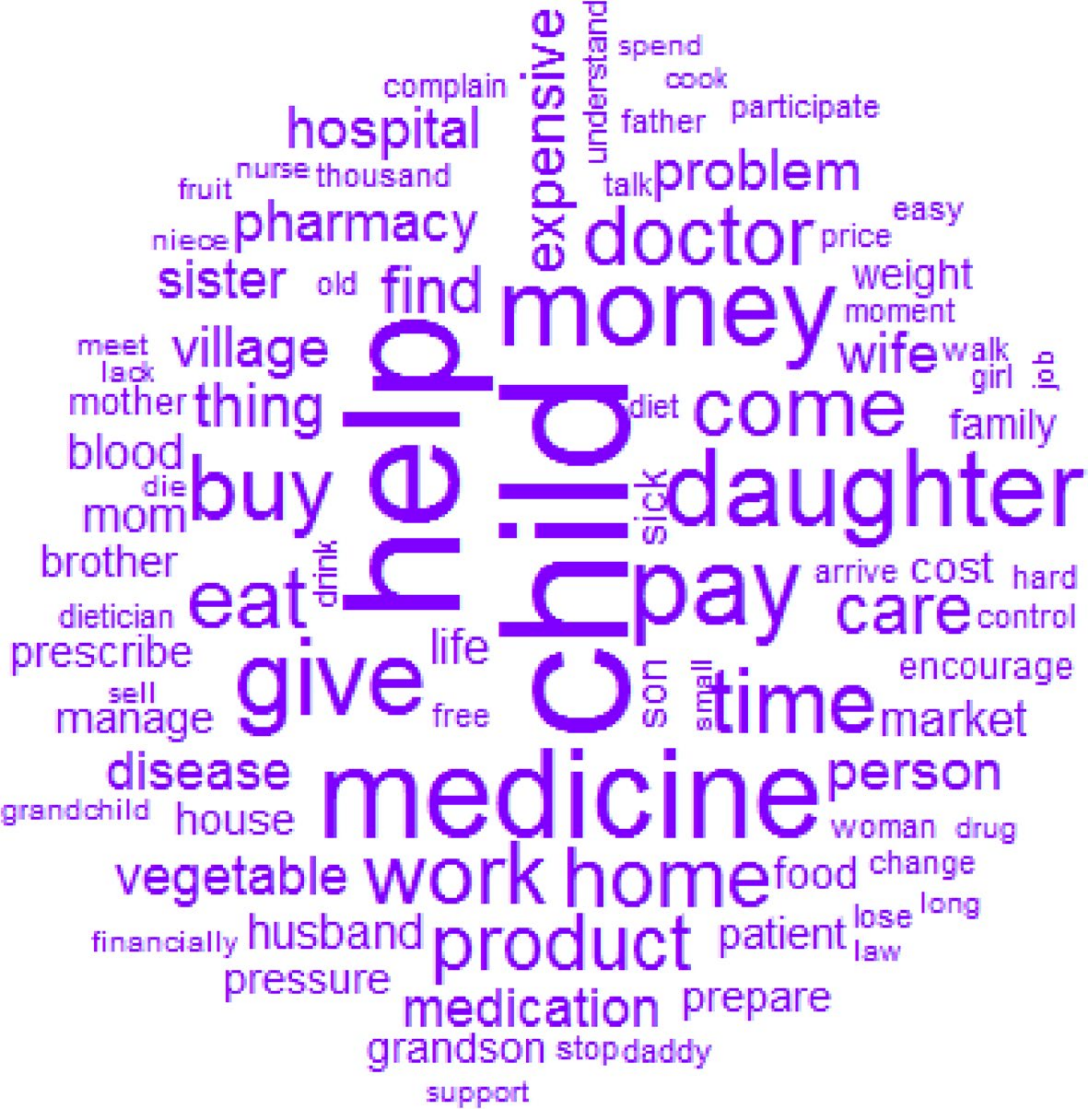
Words used to describe the resources with their frequencies correlating to their size

**Fig. 9:**
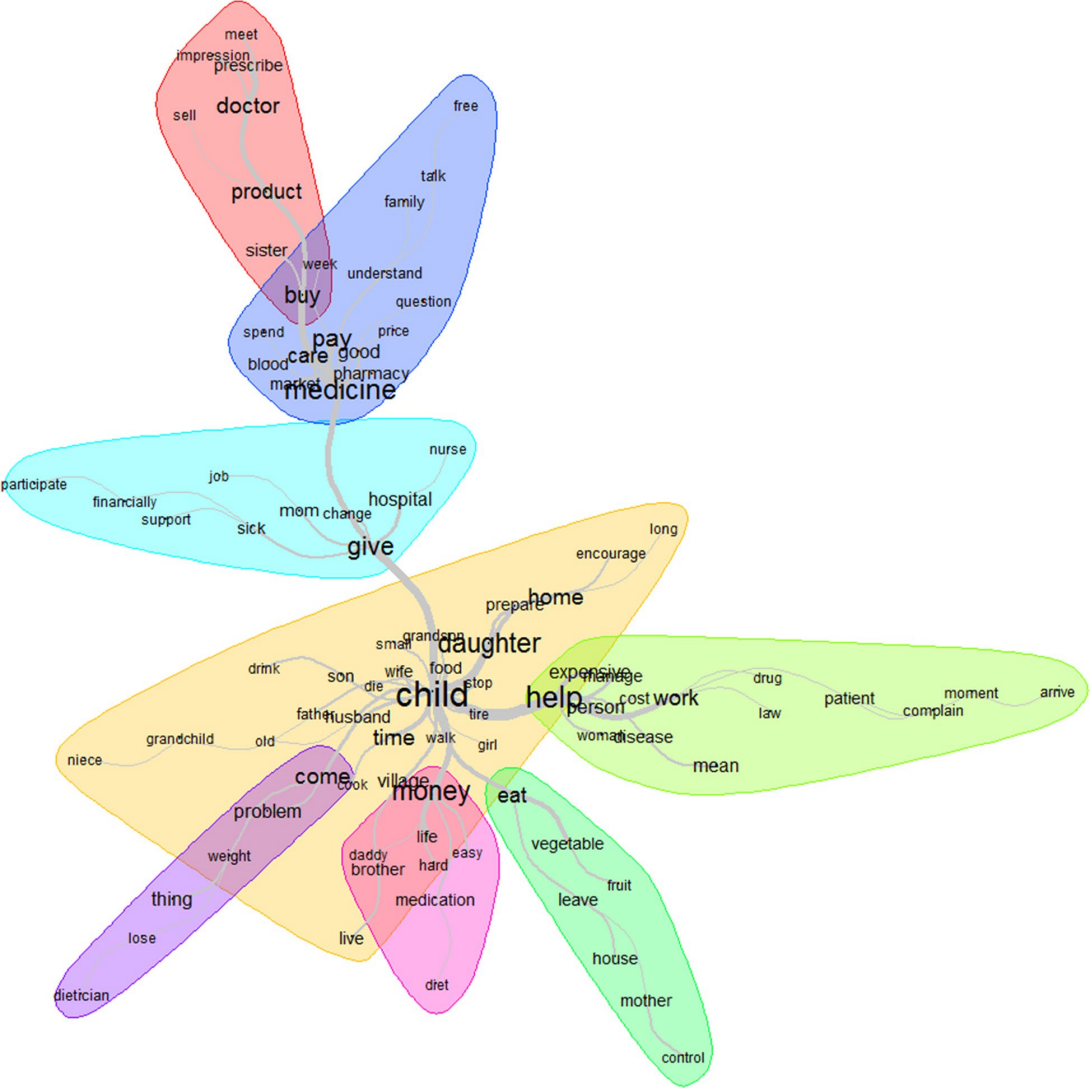
Similarities and links between the different words used to describe the resources

The top 12 words used to describe resources were: child, help, medicine, money, pay, give, daughter, buy, time, work, eat and home (Fig. 8). As patients were mainly older people, the most important resource for them was their children caring for them and providing different types of support, including financial (money, pay, buy), physical (work), material (medicine) and immaterial/moral (come, time) (Fig. 9).

Some devices were not always available when needed, being shared among several healthcare units. Being overused, they often failed to work during the consultation. The limited/small rooms for PEC and the reception were barriers for FCs and often deterred their involvement in their patients’ follow-ups. Medications and tests were usually out of stock, which affected adherence to treatment.

“… Her hypertension medicines became rare and we searched for almost 2 months and couldn’t find anywhere… when we came back to see her doctor … she prescribed another medicine … There's often when you arrive the product to measure BS is out of stock in the hospital…” (FC8)

The presence of several SPs facilitated PE and FCs who appreciated the subsidy of certain products and services, and reported good follow-ups.

"I've been here since 2002…This is my 3rd patient book …I'm here …every month.” (P10)

Patients reported being financially sole responsible for their disease as a barrier to treatment adherence. Having a family or community support or be followed up by a dietician was a facilitator of treatment adherence.

“…as I was followed by a dietician the weight dropped constantly.” (FC16)

### Belief

Believing that their disease is incurable, caused by spiritual forces, or from a curse, hindered patients’ treatment adherence.

“…I am believing that there is a person who is the cause of my disease … I think bad spiritual forces have fallen on me, I’m the unlucky one to take that disease.” (P6)

In contrast, believing in God was empowering for patients.

“… I thank God…people will give glory to God about my illness in this hospital… all my fears disappeared…” (P21)

### Satisfaction

Finding satisfaction with life was empowering for some patients’ adherence to treatment. Patient-SP interactions contributed to patient satisfaction.

“…I'm fine with the doctor, when I'm with him, he gives me advice and recommendations, he supports me a lot, so the doctors are good here. We are very lucky here as patients…” (FC10)

### Findings from observations and documents

The 29 observations of consultations of patients by SP in their offices and seven observations of care received by inpatients from GP in their hospitalization rooms, plus the nine documents, and field notes analyzed led to concordant findings with the interviews. This helps us to overcome the limitations of each source, thereby limiting the biases of distortion or limited framing of perceptions. The particularity of the observations was mainly related to the individual and unstructured nature of the encounters with the health professionals, with few minutes of passive interaction with the patients and their families. This was in line with the documents analysis, which revealed the absence of a standardized protocol to guide the provision of health care to patients in the hospital, limiting the process of PE. The documents analysis also revealed a lack of patient empowerment strategies in the national action plan against non-communicable disease, including funding constraints and high degree of centralized lines of control and complex administrative procedures with poor management and leadership in the hospital. The lack of health insurance identified through the documents analysis was directly related to the financial barrier identified by the patients and families.

## Discussion

The purpose of this study was to uncover, from participants’ experiences and perspectives, barriers and facilitators associated with patient empowerment for self-managing hypertension and/or diabetes.

The misalignment of strategies adopted at the central/ministry level of the healthcare system with the management of the disease at the organizational/hospital level and the socioeconomic conditions of patients were the greatest impediments to patient empowerment. The funding constraints and hierarchical bureaucratic structure of the healthcare system in Cameroon, characterized by a high degree of centralized lines of control and complex administrative procedures, were major obstacles to the development process of PE. Similar findings were reported elsewhere in Africa [39, 40] and agreed others which identified the bureaucratic top-down structure as barriers to effective implementation of PE strategies for chronic NCDs [14, 41].

The main facilitator at the hospital/organizational level was the patient education counselling with different products and services to educate and follow-up patients with hypertension and diabetes. But the time accorded to each patient by some healthcare providers was not enough to share information about the disease and associated factors that can increase a patient’s desire and ability to participate in the decision making process, as also found elsewhere [41, 42].

FCs played an active role in the development of patient empowerment by seeking information about their diseases and recommended treatment. This role is under appreciated in the framework of chronic care model and self-management of NCDs in resource-limited settings [43, 44]. Patients make best decisions when equipped with accurate and clearly communicated information with available space to explore options and choices [45].

The healthcare delivery at the hospital was hampered by long waiting times to see a specialist, the poor quality of service and overuse of some devices [44]. Poor management and leadership in healthcare as well as the context of care often led to corruption, disrespect, command, or mistreatment of patients and their families in the hospital system. Similar findings have been reported elsewhere in Africa [16, 46]. Healthcare professionals being key contact points between patients-families and healthcare systems, they often caused patient disempowerment as other studies have found [47, 48].

The frequency of follow-up and the quality of patient-specialist interaction emerged as empowering for patients, as found in other LMIC settings [44]. Unfortunately, the hospital healthcare in Cameroon remains largely an acute care model with perceived staff shortages and ineffective communication, undermining the potential empowerment of patient and FCs. The relationship between patients/FCs and healthcare professionals should be more frequent with a reciprocal process of mutual respect for each person’s knowledge and the right to make informed choices [20, 48], in a partnership approach seeking to balance clinician’s expertise with patients’/FCs’ preferences. Available evidence suggests that health outcomes are better when patients are more involved in decisions about their treatment [45, 47, 49].

The fear created by some healthcare professionals about the consequences of the disease deterred patients who felt disempowered to take action [48]. Conversely, trusted relationships reassured patients that their health will improve. Healthcare providers involving patients and families in the treatment decision making process tend to stimulate patients’ motivation and engagement in the healing process [48].

Losses of loved ones and thinking that there were no solutions stalled patients’ engagement in the empowerment process because they considered their situation out of control. Meanwhile, some patients were motivated to control their disease by replacing old habits with new ones favoring recovery. SOC being an interaction between the individual and life context [26, 27], feeling supported contributed to patients’ motivation in activating and making the best use of their inner assets, which increased their confidence in their ability to manage their condition.

GRR in terms of medicines, equipment, laboratory supplies and personnel are needed for delivering good healthcare. Barriers identified at the hospital/organizational level included the unavailability and/or misuse of resources. The budget allocated to managing NCDs in the healthcare system in Cameroon is awfully low [50], given the 15% of the national budget recommended in Abudja in 2001 [40]. The lack of health insurance makes patients and families bearing treatment costs in Cameroon, an enduring situation in LMICs [9].

Two-third of patients were women, of whom about half were widows with husbands who died from these diseases or their complications. Over two-third of FCs were women, due to gender norms in Cameroon and elsewhere in LMICs where women remain the primary family caretaker [51].

The influence of belief was positive or negative on patients’ empowerment, depending if it was motivational or not to the development of patients’ autonomy to self-manage their disease. Believing that the disease was caused by spiritual forces or curse did not allow patients to change their lifestyle which plays a key role in NCDs development, occurrence, and complications; believing in God motivated patients to follow the therapeutic plan. Our findings support previous research showing that health belief plays a role in patient decision through the process of healthcare service [52].

Considering patient satisfaction as the patient’s positive evaluation of healthcare dimensions, patient experiences with medical staff were influential factors. Patient satisfaction being an indicator of the quality of services [53], the more patients are satisfied the more they will be committed to follow-up services and then develop their ability to self-manage their disease and vice-versa.

### Trustworthiness/rigor

Four relevant criteria to qualitative research were considered to increase scientific rigor or trustworthiness (confidence in the accuracy of results): consistency, transferability, reliability and confirmability [36]. To check for consistency, data sources were triangulated by seeking different data sources in the study, and by crosschecking different points of view of stakeholders. This study was limited to one of the main hospitals in Yaounde, Cameroon; results may not be generalizable across all contexts. However, we have provided abundant description of the context to allow readers or users of the research to judge potential transferability to other contexts. To ensure good reliability, various measures were taken: (i) the development and application of a conceptual framework of analysis whose central concepts were clearly described as well as the methodology; (ii) the verification of the thematic codes by the co-author; and (iii) the exploration of the concordance of the results using triangulation. The confirmability potential of this study lies in the fact that the other three criteria have already been met. Thus, the integrity of our results is rooted in the corpus of data, in the detailed description of the method used (sequences of data collection, analysis, and transformation), and in the entire validation process we followed.

### Strengths and limitations

A major strength of this study is its multilevel approach to studying patients’ and FCs’ experiences and perspectives representing various socioeconomic and cultural backgrounds, with different data collection methods and pertaining to all three levels of a national healthcare services. Another strength is that patients and FCs had unique experiences that informed their opinions on what happened in the national healthcare system; considering patients’ and families’ recommendations about structural, broader and systemic changes may promote successful patient engagement and outcomes. Finally, these unravelled experiences and perspectives are invaluable to filling missing healthcare gaps in resource-limited settings like Cameroon.

This study was limited to one of the main hospitals in Yaounde, Cameroon; results may not be generalizable across all contexts. However, they may be helpful to the extent they are transferable to similar settings.

## Conclusions

This study aimed to pinpoint influential factors associated with the development of PE in the primary healthcare context of Cameroon. Our multilevel investigation of PE in the management of hypertension and diabetes found that patients-identified factors mainly related to self-management of the disease while caregivers focused chiefly on factors at organizational/hospital and central levels indirectly related to disease management and beyond their control. The preponderance of patient-level factors compared to those at central/ministry and hospital/organizational levels calls for the need to pay more attention to them in resource-limited settings. Accounting for patient’s and families’ experiences and opinions may be key to improving healthcare delivery.

## Supplementary Information

**Additional file 1.**

## Data Availability

All data generated or analysed during this study are included in this published article [and its supplementary information files].

## Abbreviations

FC: Family caregiver
FGD: Focus group discussion
GP: General practitioner
GRR: Generalized resistance resources
LMIC: Low- and middle-income countries
NCD: Noncommunicable disease
PE: Patient empowerment
PHCDH: Primary healthcare district hospital
QDA: Qualitative Data Analysis
SOC: Sense of coherence
SP: Specialist physician
