# Supplementary material for: Patients’ and family caregivers’ experiences and perceptions about factors hampering or facilitating patient empowerment for self-management of hypertension and diabetes in Cameroon
Source: BMC Health Serv Res. 2022 Nov 21;22:1381. doi: 10.1186/s12913-022-08750-4 (PMC9677916; doi:10.1186/s12913-022-08750-4)
Supplement: Supplementary file 1 — Additional file 1. [file 12913_2022_8750_MOESM1_ESM.pdf]

Supplementary information files

Additional file 1. Interview guides

Interview guide for patients

Identification:
- Date.........; time:………… beginning..........., end...........
- Health center:
- Name and surname:
- Education:
- Occupation:
- Marital status:
- Age: ………. Sex: ……….
-Number of years with the disease:

1) What can you tell us about your disease? Do you have a treatment plan prescribed by your physician or any other healthcare professional?

➢ Causes and prevention of the disease, therapeutic plan, diet, physical activity, quality of life, etc.

2) What can you tell us about the patient empowerment approach?

➢ Development of sense of coherence (intelligibility, manageability, and meaningfulness), resources (internal, external), components, adherence, etc.

Pause for clarification if necessary ……………

3) For you, how does the national program for chronic noncommunicable diseases in general and for hypertension/diabetes in particular allow you or not (facilitators or barriers) to be empowered, to self-manage your disease, to adhere to your therapeutic plan and to achieve the best health outcomes?

➢ Planning: strategies, objectives, allocation of resources (material, financial, human, infrastructure, etc.), subsidy of products and services, etc.

4) For you, how does the organization of services and healthcare in the hospital allow you or not (facilitators or barriers) to be empowered, to self-manage your disease, to adhere to your treatment plan and to achieve the best health outcomes?

➢ Availability, accessibility and use of resources, quality of services and health care, patient education, etc.

5) For you, has the direct care or the practice of healthcare professionals allowed you or not (facilitators or barriers) to be empowered, to self-manage your disease, to adhere to your therapeutic plan and to achieve the best health outcomes?

➢ Understanding, listening, quality of relationship with healthcare professionals, etc.

6) For you, which factors (facilitators or barriers) within your social network (family, friends, community members) do you think help you to be empowered, to self-manage your disease, to adhere to your therapeutic plan and to achieve the best health outcomes?

Interview guide for patients’ family caregivers

Identification:

- Date.........; time:………… beginning..........., end...........
- Health center:
- Name and surname:
- Education:
- Occupation:
- Marital status:
- Age: ………. Sex: ……….
- Number of years (months) spent with the patient in the management of the disease:

1) What do you know about your patient's disease (hypertension/diabetes)? What do you know about the treatment plan prescribed by your patient’s physician or any other healthcare professional?
   - ➢ Causes and prevention of the disease, therapeutic plan, diet, physical activity, quality of life, etc.

2) What can you tell us about the patient empowerment approach?
   - ➢ Development of sense of coherence (intelligibility, manageability, and meaningfulness), resources (internal, external), components, adherence, etc.

   Pause for clarification if necessary ……………

3) For you, does the national program for chronic noncommunicable diseases in general and for hypertension/diabetes in particular allow or not your patient (facilitators or barriers) to be empowered, to self-manage the disease, to adhere to the prescribed treatment plan and to achieve the best health outcomes?
   - ➢ Planning: strategies, objectives, allocation of resources (material, financial, human, infrastructure, etc.), subsidy of products and services, etc.

4) For you, does the organization of services and healthcare in the hospital allow or not your patient (facilitators or barriers) to be empowered, to self-manage the disease, to adhere to the treatment plan prescribed and to achieve the best health outcomes?
   - ➢ Availability, accessibility and use of resources, quality of services and healthcare, patient education, etc.

5) For you, does the direct care or the practice of healthcare professionals allow or not your patient (facilitators or barriers) to be empowered, to self-manage the disease, to adhere to the prescribed therapeutic plan and to achieve the best health outcomes?
   - ➢ Understanding, listening, quality of relationship with healthcare professionals, etc.

6) For you, which factors (at the individual, family, community levels) do you think promote or not your patient's empowerment, adherence to the prescribed therapeutic plan and to achieve the best health outcomes?

Additional file 2. Barriers to patient empowerment in the management of hypertension and diabetes along the three levels the healthcare system in Cameroon

Health system	Mega codes (9)	Sub-codes (22)	Themes (53)	Representative quotes
**Central level (4)**	Sense of coherence (SOC) (3)	Intelligibility (2)	1- Centralization of health system and not patient involvement in the implementation of national action plan against chronic NCDs	1- “About the health ministry ... I can't know what's going on there so I can't talk too much about the national action plan against chronic NCDs because I don't know. For programs, I'm not aware. It’s when I was walking along the road, I saw screening test campaign, when I arrive there, I am told that the date had already passed.”
			2- Lack of knowledge of national action plan against chronic NCDs (Diabetes/HBP) by patients and families	2- “I don’t have any idea about the health ministry program.”
		Manageability (1)	Awareness strategies are not adapted to reach as many people as possible	"I've already heard about that I think on the radio, ... but not everyone has radio or TV. … The government needs to talk more about it in neighborhood churches and meetings... I think it is that the ministry must continue to strengthen all these strategies, not only in hospitals, but also in the associations of the neighborhood, he has to send members to talk to them ... they should train people who give us information from neighborhood to neighborhood through flyers too.…they have to send the text messages in our phone... it takes advice from the ministry of health that we have to do sports or walk regularly, do not smoke or drink too much alcohol ... So, I think it takes this to raise people's awareness, we don't need the advice only when we come to the hospital.”
		Meaningfulness	/	/
	Generalized resistance resources (GRR) (1)	Internal resources	/	/
		External resources (1)	Low budget allocated to the management of chronic NCDs, no subsidies for products/services of diabetes/HBP (The ministry of health favours interventions against CDs more than chronic NCDs)	“... because sometimes the products are so expensive for patient and his family ... For example, I spend at least 25000 CFA francs a month to buy my medicines, and it is for life. If the government can pay at least half of the total cost of health care, it will be so helpful."
	Health outcomes	Adherence and health outcomes	/	/
		Beliefs	/	/
		Satisfaction	/	/
**Organizational level (19)**	SOC (8)	Intelligibility (3)	1- Not all participants are aware of the existence of education club for	1- “No, I'm not aware of that, so they help people who have hypertension and diabetes? I'm not aware of it. I haven't been told about it since I've been here ... No, I do not know, every time when we arrive, we see the doctor and we left. I had no information about the club.”

			diabetics and Hypertensive patients	
			2- Lack of communication /explanation between doctor and patient /family about their illness	2- "… The doctor didn't tell me anything, you know you doctors know what's wrong, but you write it down in my notebook. So, it's not worth asking the questions, I just want to be cured, be in good health." "… Another difficulty is that, I feel that the relationship is only between the doctor and the patient. So, the family doesn't have a right to talk. As for example, my husband went there once to ask clearly, he told me that he did not have to talk with the doctor. I personally have never been able to talk with the doctor. So, she's the only one who talk... when we arrived, they welcomed us, and ask me just to know her situation, I say she is diabetic, that all."
			3- Doctor does not clarify the recommendations to be followed to patients	3- "... The doctor had asked for the first time that she had to do the sport. We understood sport like bring her to a gym … (laugh), because we registered her in a gym and pay. They make her workout like if she was a girl, and she got sick after she left to a single session. It was too intense for her. (The doctor didn't clarify that workout for her means walk)"
		Manageability (4)	1- Unstructured reference /counter reference system	1- "... because where I was in Etoug-Ebe there (health centre from another area), the nurse says that Mama go to BHD because she knew that maybe it was high blood pressure, she tells me that, no Mama we cannot manage here...."
			2- Waiting time to see the doctor	2- "… We arrive at 9am, she's diabetic who can't eat everything. So, according to her diet, she cannot eat here, also because she had a health problem this morning. She will first see the Doctor. But if from 9am, she finally sees the doctor around 2pm it is not easy. So, it's at the reception, I don't know how they are organized, I don't know why it is so slow, sometimes we arrive we put our notebooks and they reverse everything, the first become the last."
			3- Malfunctioning of some devices	3- "…even the device to measure blood pressure, as an automatic device doesn't work sometimes, maybe because they used it a lot. But by chance, it often happens that in the group (of patients) there are women who have their individual devices, so, they propose to the nurse to use it to help the rest of patients... also, sometime we arrive here and the device to measure the weight is not working. For e.g., you measure your weight it gave you a weight that was not compliant, it bothered us too much, we sometimes complained, because it gave you 90 kg or 100 kg while your normal weight is around 70 kg."
			4- Corruption: sale of medicines by some healthcare professionals	4- "… We don't want nurses tell you to buy the drugs they have. They tell you, come I have drugs you want while you want to go to a pharmacy… so, we don't want corruption here, after the doctor give you the list of medicines, we want to feel free

				to go where we want to buy it, not that someone (nurses) start telling you that I have that medicines here come to buy it here, it's not good like that."
		Meaningfulness (1)	Create more fear than confidence in patients	"He asked me to take the medication after the visits... he told me I have to take the medicines until I die, and I'm really scared for my case."
	RRG (9)	Internal resources	/	/
		External resources (9)	1- Skills of some healthcare professionals are questioned	1- "… but also about the exams, when someone asks to take the exams for almost 300,000f cfa, that is not normal. It feels like the doctor doesn't know what's wrong, how can a doctor prescribe so many tests, it means that the doctor doesn't know what he's doing. And when all the tests come out negative, it means that the doctor gropes, that he is overwhelmed by the disease .... So, I think that in the Cameroonian context, doctors must be precise. But I think that the problem here is people are not serious. I want to talk about professionalism... because she had a lot of drugs, really I can't figure it out, it's too many drugs."
			2- Out of stock of certain drugs/products in the system	2- "… his hypertension medicines became rare and we searched for almost two months and couldn't find anywhere... but when we came back to see her doctor here…I told her that I can't find it anywhere it's missing anyway it's out of stock on the market for almost two months. She prescribed another medicine and she finally begins her therapeutic plan." "... There's often when you arrive the product to measure blood sugar is out of stock in the hospital, this happens more often."
			3- Side effects of certain drugs	3- "…there was a product that didn't work with me here it was in 2017, this situation makes me afraid when I'm sick and come here because the product was not really adapted to me, to the point where I could die."
			4- Uncontrolled street drug purchases	4- "… I use to pay my drugs at the Mokolo market (street drugs purchases) because you know they have a big market of drugs there, but as I am now here I pay everything at the hospital pharmacy; of course, the pharmacists if they have no drugs they go to Mokolo too…"
			5- Physical structure of the hospital	5- "… The size of the reception area, the consultation offices, the hospitalization room, the space for patient education are small according to the numbers of patients ... we have to rebuild the hospital with a large room but what we have there now I think is well managed. I think that this hospital at first didn't anticipate that it would become a big hospital like this."
			6- No availability of some material resources for diabetic /hypertensive patients	6- "… What bothers me here is that, sometimes they say that there is not the machines to measure her blood sugar and blood pressure, because all the people use the same two machines there, so, when someone else is using it, we need to wait... For example the blood pressure monitor, sometimes the nurse can be measuring your blood pressure and someone else comes and takes the device."

			7- Not enough human resources (nurses) for diabetic /hypertensive patients	7- “I think that the hospital could try to increase the number of nurses, especially the days they know that there is the diabetics club. Because when we come from our houses, it is too early, we did not eat, and we spent a lot of time here because they were just two nurses for all of us. So, I think that we can increase the number of nurses at the reception so that things go faster, so that we also go home. When you stay there from the morning until noon you didn't even have breakfast it's not good."	
			8- Recommended therapeutic plan and diet are very expensive	8- “And for the diet, she said that the diet and quite difficult, it is quite expensive, she also says that reconciling the diet and the drugs is extremely expensive, and sometimes she can’t take the drugs when she wants, because as I told you, it is extremely expensive, to the point that she is most of the time with money problems.”	
			9- No financing of products / services for diabetic / hypertensive patients	9- “… because the financial problems do not allow us to always follow our diet as we should and then I also deplore the fact that HBP drugs are also too expensive in Cameroon. I believe that the government should help hypertensive patients, they can even finance their drugs. Me for example I pay almost 20,000f cfa for drugs, I have two products that I take differently in the morning and evening at bedtime, for me I think it is extremely expensive and it is almost the same for all Cameroonians anyway, it's really expensive, if only the government could do something for us it will be so welcome.”	
	Adherence and health outcomes		/	/	
	Beliefs		/	/	/
	Satisfaction (2)		1- Poor quality of reception of patients by the nurse receptionist	1- “... she told me today that based only on the quality of the reception, before she sees the doctor, she would have already abandoned the hospital…, since the one who measure the parameters at the entrance there, he threatens people, he behaves badly with people ... she told me that the guy who is at the reception there, when she only thinks she's going to go over there and he's going to measure her parameters she's already traumatized.”	
			2- Difficulty meeting the doctor	2- “… your doctor from Douala who takes care of me here knows that I came back from the USA, I have not seen him yet, I do not know if I will see him there today, it is him who followed me since I came here even before I travel. He has not yet had time to prescribe something for me that is all. So, as I have been here since Thursday, I'm just saying that maybe... there's a girl who told me he'll come today. I only know it's good if he'll be here today.”	/
**Individual level (30)**	SOC (14)	Intelligibility (5)	1- Low level of education (mainly for older people)	1- “… I know that she can be blocked at some level because of French language. They say things that she doesn’t understand, but if they explain slowly, as to older mom who didn’t go to school, she told us that she will understand few words or more.”	

			2- Poor understanding of doctor's recommendations	2- "… I don't have to eat vegetables every day to heal."
			3- Diseases usually diagnosed late or when hospitalized for another disease	3- "I think she had been sick for long, she went to the hospital, that's when they measure her blood sugar to find out, to realize that she was sick... For her, the doctors told us that it was already late, it was already at the advanced level ... doctors through the exams realized that she has diabetes."
			4- Does not try to understand his diseases and thinks it is doctor's responsibility	4- "I'll just tell her that the main thing for me is to heal, if she wants to change something in my therapeutic plan, she is the Doctor, it's she who knows the treatment if she can do anything as long as I find healing there's no problem."
			5- Lack of Knowledge about diabetes/hypertension	5- "I have no knowledge, what I know about diabetes is that it is a disease, it is harmful, it makes you have pain in the body and sometimes you find yourself comfortable as if you do not suffer from anything. I do not really know how to explain but it is a disease that only you who know that you are sick."
		Manageability (5)	1- Sedentary lifestyle	1- "I'm at my daughter's house, when I'm there she doesn't even want me to go to the village, ... I do not even work there, I get up in the morning they give me food, during the day they give, in the evening they give, so when I am not eating, I am sleeping. I do not work [anywhere]."
			2- Changing living environment	2- "… here in Yaoundé she does not have this kind of activity, it is in the village that she has the activities at the church, in the women's associations in the village. But really when she is here in town she has no activity and precisely when she is in the village with the associations she is obliged, she always moves there, she remains active but when she is here she has no activity except to go to visit her nieces, her daughters but no more than that, she does not have more here in town."
			3- Discomforts caused by changing lifestyles	3- "... for almost two to three months, yes she said that she had to restart with the walk, well she could quickly do it when she said that she walked maybe two or three times, but now she got tired and have dizziness every time, which doesn't allow her for being able to walk. Otherwise, when she had started, regularly she did it minimum once a week, max three times."
			4- Not liking the taste or not having the appetite for recommended healthy foods	4- "… I'll tell you why, I don't like vegetables like salads and the others. Now, I do not have appetite. But I avoid things like veggies, and I have a lot of cholesterol, that's why I don't like bread too … Because I don't eat well, I hate food, the medication is helping me very well."
			5- Stress caused by external/internal factors	5- "First there's the fact that she's not following the diet properly and there are a lot of elements. I think there are a lot of external factors that cause her to experience intense moments of stress so, there are all these factors that make that at some point she no longer controls herself properly."

		Meaningfulness (4)	1- The loss of a loved one / No more reason to live	1- "… But as I just lost my husband in May, and my blood sugar and blood pressure have started to rise again… I have no reason to keep fighting for life."
			2- Focusing on family problems / Not being positive	2- "I have my boy who is with his wife here, they have problems at any time now, they broke up, they are no longer together, but my son comes home being drunk all the time, when he comes, he talks, he talks, it does not stop or leave me and it tires me, this makes my blood pressure increases."
			3- Don't believe in recovering process	3- "... I believe that when this thing here (diabetes) starts on someone it doesn't end."
			4- Don't take therapeutic plan seriously / lack of motivation	4- "It's difficult. As I tell you, I play with it, that is why my blood sugar is not stable. If I really followed the recommendations normally, my blood sugar would be stable. Like this morning it is 1.40. ... even the beef or pork I eat, I'm told the beef is not good as it's red meat, but we do not find it every day so when I have occasion, I eat, that's how I live. Oil doesn't even like me, salt and sugar too, … I killed myself because someone can understand and not respect, do you understand me, that I do not respect it is not good and I have already become accustomed to do like that."
	GRR (4)	Internal resources (2)	1- Difficulty changing / keeping healthy lifestyles	1- "… it's not even easy, it's a fight, it's really complicated, she didn't accept it, especially the very complicated diet, she's rather attracted to the products she's asked to avoid, so really it hasn't been easy it's even a fight because so far it's not easy."
			2- Lack of determination / laziness	2- "… I already have the sports outfit, but I haven't started yet the sport, every time I say I'll start tomorrow, and when the morning comes, I keep sleeping, it's like that. It's laziness I can't tell you more is laziness."
		External resources (2)	1- Lack of help/support or being solely responsible for his disease	1- "My problem is I'm the head of the family. I'm a dad of a few children and alone. When I'm sick and when it comes to buying the drugs, I've been hospitalized for quite a while and the drugs and exams and everything and everything is looking for me. When you make efforts to find money, to feed your family, it takes a lot to fight for a lot of products so it's difficult. I want to talk about these raw vegetables and then foods when you leave the hospital you have to change the lifestyle, the diet to get back in shape in few days."
			2- Lack of financial to purchase recommended medicines / healthy foods	2- "… She also says that reconciling diet and medication is extremely expensive, and sometimes she can take the medication when she can, she told me she juggles... But it can happen once a month that she eats a food that is not recommended for her like couscous of corn, rice... things like that."
	Adherence and health outcomes (7)		1- Give up the decision of not consume un-recommended products	1- I can take my ISENBECK (beer), but I do not abuse, as on the party day, I said that weeeee, even God cannot punish me, so I take two beers … I take one in the morning the other in the evening, it is enough for me after I continue with my "Supermont" (mineral water)."

		2- No positive impact of adopting healthy lifestyle on health outcomes	2- “As I see the diet does not work, the diet does not influence because when she starts her diet, the kind of diet with no oil, she takes a lot of soup, and raw foods, but from time to time we realize rather that her blood sugar and pressure increases, really it's not the diet, I can say that it did not have an influence by following the diet, it did not have an influence on her diabetes.”
		3- No positive evolution in the treatment of the disease	3- "At 57 she's still young, I don't understand why it's so difficult to heal from this diabetes, because I know [people] older than her, suffering from it, but not feeling as much pain. She sometimes cries, she screams, it doesn't evolve, it's serious.
		4- Poor health / Poor physical condition	4- "For sport, now I can't do the sport because walking is still difficult for me and the movement is difficult, first I have to get healthy. When I get healthy and know that I can walk well and the hand is already working well, I'm going to start again with the sport."
		5- Improvement in the patient's health	5- “… and also when she feels a little better, she stops with the drugs … and when she feels it's coming back (the disease), she restarts taking medication.”
		6- Comorbidities / Complications of the disease	6- “I feel like it's diabetes that causes other diseases, because I have many problems: diabetes, high blood pressure, stomachache, nerve problem, she has nerve problem…But I think that diabetes is the cause, everything starts with this.”
		7- Wait until the disease worsens before going to hospital	7- “My son had had the same symptom, so he told me he put salt in the yogurt and drink, I said that's what calmed you, he said yes. I also started to take this, two days after, I saw it did not work. I go to see another home doctor in the neighborhood, he gave me the injections, he says it's going to be okay. We were already Saturday. I went to the church and back, I felt how it was wrong, my wife said no according to how we spent the Saturday night, she cannot accept that we stay at home like that, she proposes to go to the hospital. We decided to come here... and after exams they say I cannot go back because my blood pressure is too high. So, they kept me here and since then I say that things already get improved, compared to last night I slept quietly at night.”
	Beliefs (5)	1- Believing that the disease is caused by spiritual forces or a person	1- “… sometimes when you are sick, you can believe that there is a person behind. I am believing that there is a person who is the cause of my disease. .... I think bad spiritual forces have fallen on me, I’m the unlucky one to take that disease.”
		2- Believing that the prayer is enough to recover health	2- “... I prayed God that one day I recover health, so the disease would no longer bother me. God himself will operate the miracle, so that this high blood pressure will disappear.”
		3- Believing that alternative / traditional medicine is the best solution	3- “… she takes a lot of traditional / natural products. When people tell her that try to take this, it's going to help you, she puts more emphasis on those things."

			4- Believing that following one good action is enough to recover health	4- "… I thought that drinking a lot of water every day early in the morning, fasting, like a therapy will heal me, but so far it has not given good result."
			5- Believing that there is no treatment, that it's an incurable disease	5- "... I think that if the disease comes, you're going to die with that, there's no solution, is that you're going to pass. ... It's for life because even my father suffered from this until his last day. I already know that they are always saying it's an un-recovered chronic disease."
		Satisfaction	/	/

Additional file 3. Facilitators of patient empowerment in the management of hypertension and diabetes along the three levels of the healthcare system in Cameroon

Health system	Mega codes (9)	Sub-codes (21)	Themes (40)	Representative quotes
**Central level (2)**	SOC (2)	Intelligibility (1)	Organization of free awareness/counselling or screening campaigns	"... The ministry often organizes awareness campaigns or screening campaigns; it is at this kind of free campaign that my husband was diagnosed positive to high blood pressure."
		Manageability (1)	Using different means/canals for awareness/counselling	"... We often hear about the diseases on the radio, often on the TV, there are often posters along the road or even in the hospital here."
		Meaningfulness	/	/
	GRR	Internal resources	/	/
		External resources	/	/
	Adherence and health outcomes		/	/
	Beliefs		/	/
	Satisfaction		/	/
**Organizational level (12)**	SOC (5)	Intelligibility (3)	1- Presence of education club for diabetes and hypertensive patients at the hospital	1- "… when we come here, we are asked not to cross our arms, but to be active, because by not doing anything, that is what increases your pain. I think organizing club like that is good, because there are a lot of people who live without knowing how to do it and if there are clubs like that to give advice, I think it helps people a lot, they will comment on the state of health, will know their body and know how and what to do to take care of it."
			2- Distribution of education sheets on healthy lifestyles (foods choose, foot hygiene, etc.) to patients	2- "… he had been given a sheet and there was everything he had to eat in large quantities, what he had to eat moderately and what he should not even taste... what he was no longer supposed to eat at all was on the sheet, it is the peanut that he should no longer eat, alcohol, salt…"
			3- Explanation of the disease and treatment plan to patients and families by medical specialists	3- "I was feeling it difficult to manage it home until I came here…, for me it was not easy before. Because I lost my breath, so I was really worried, I ask myself, is it spiritual? So, when I met this doctor, he explained that it is just a disease, it is not spiritual. So, that one explains, consult me very well.""I understand very well because he takes the time to explain well, and when I do not understand I ask him the question again and he starts again until I understand and at home I explain well to my mom."
		Manageability (2)	1- Patient follow-up: frequency, duration, and nature of visits	1- "It really helps since the place is already suitable. She comes frequently, when she arrives, they take time to measure her parameters. They control her blood sugar, her weight, her blood pressure and all the arrangements are made so that she can be comfortable in addition to the diet to which she is subjected… there is a good follow-up

				and I do say thank you for that. She's well followed here."
			2- Relationship of trust between doctor-patient/family that facilitates mutual understanding	2- "This Doctor has been my doctor for 5 years. I already talk with him as if I were chatting with the child that I gave birth myself, he is patient, he listens and he gives advices... he explains very well... as I say, every time I come I don't hide anything, like this time, my blood sugar and blood pressure have increased, and I reduced everything I ate and everything I drank like the baobab juice I drank yesterday."
		Meaningfulness	/	/
	GRR (3)	Internal resources (1)	Ability to retain patients	"I've been here since 2002 when we left the central hospital. At the same time, they created this club (PEC) with a great ability to retain patients. This is my 3rd patient book here, I'm here in the hospital every month."
		External resources (2)	1- Availability of medicines/products/services at a lower cost	1- "…about pharmacy, there is no comparison between hospital pharmacy and the outside pharmacies, the products are cheaper here in the hospital. … 95% of the medicines can be found here and the rest it's in other pharmacies." "…the club here is good because in the village when they measure our blood sugar it's a thousand francs or two thousand while here the diabetic club takes us five hundred francs."
			2- Presence and availability of different types of health care professionals: Nurses, general practitioners, and diabetes/HBP specialists	2- "... because where I was in Etoug-Ebe there, the nurse says that Mama go to BHD because she knew that maybe it was high blood pressure, she tells me that no Mama we cannot manage here…" "I believe that in terms of availability the nurses are always there when you arrive at the hospital, the doctors come according to their plan, and they are many here."
	Adherence and health outcomes (1)		Better adherence and health outcomes	"They advise him every time we go to the hospital; the doctors talk to him about his diseases, and this motivated him to leave a lot of bad things and follow his treatment well... I see how things have evolved and it's getting better and better."
	Beliefs		/	/
	Satisfaction (3)		1- Cleanliness at the hospital	1- "... there is already a lot of improvement, I admire this hospital first because of its cleanliness compared to elsewhere is what attracts me first here, the toilets are well the reception is impeccable so that's really what attracts me and then I have no problem since I come to this hospital I'm welcomed, I cannot complain about anything."
			2- Reception and interaction of healthcare professionals with patients and families	2- "... my doctor is a smiling, welcoming young guy, he hugs me, he even carries me, he gives me courage, if he wants to examine me he gives me the courage, how I have to get on the table and he doesn't act with

				brutality like some doctors do. No, for example, here he says dad gets on the table we must examine you. For me, the welcome is very warm."
			3- Dynamism/energy of the PEC-T2D	3- "… She participates in education groups and there they give them advice, they do small exercises, they even make them first dance before start takes measure. So they guide them by dance like little children of kindergarten, it's beautiful to see, even there are times I go there I sit down just to watch how they do the little things, it makes me laugh and happy." So frankly the diabetic club is very good comfortable with very little things."
**Individual level (27)**	SOC (16)	Intelligibility (7)	1- Use different ways to access information about diabetes/hypertension	1- "... I myself do a research, I surf on the internet it helps me know how I should behave, what I have to eat, what I should not eat, what to do to avoid this or that … this's how I live then now."
			2- Clearly communicate their needs to the doctor	2- "… she speaks French and the Doctor understands very well and when the Doctor did not understand, she says no, that is not what I meant. I wanted to say…"
			3- Understand the physician's explanations/recommendations	3- "The care is very good because when I meet the Doctor for a problem, he tries to explain to me even if I do understand, the next appointment, I ask him again and he tells me very well and I finally understand there is no problem, so everything is good."
			4- Seeking to know the health status (to be diagnosed)	4- "So on this third January, I went to a clinic, I did a test, in conclusion, the result was positive, after the doctor asked me if it was a prescription, I said no, just that I decided to do it by myself. So, they consult me, and I realized that I'm diabetic."
			5- Recognize the signs/symptoms of diabetes/ HBP	5- « ... When I found that he was peeing every second, I said what happens? It must be a disease like that, and he says, but what kind of disease? I say no it is already a disease the kind you already pee without even controlling yourself, we have to go to the Hospital ... when he came here, he told me that at first they told him it could be an infection, and after the examinations they confirmed that it was diabetes."
			6- Know the causes of diabetes/hypertension and prevention strategies	6- "It seems to me that prevention is in the diet and in physical activity, you don't have to let go because you have to move for your health, you have to do sport you have to control your diet. There is the stress also, you have to know how to put things into perspective, so you do not have to manage too much stress"
			7- Know of the expression "patient empowerment"	7- "… to define empowerment, I realize that it's like an awareness, … in our community we call it awareness. Well, for my mother this term refers to her autonomy, her state of mind in which she must live, she must follow-up her diet, take her medications

				because she is 75 years old, so she has a follow-up for life. ... she can't submit to intense sport as us, her sport can be walking from time to time."
		Manageability (4)	1- Find ways to reduce the cost of medications/products prescribed by the doctor	1- "No oh, they don't give me the discount, thing is, the bottle I was paying at 15 000f cfa, I now pay it at DSCHANG (Ouest region) at 10 000f cfa for the same quantity. I pay there because I have a cousin who is nursing at DSCHANG, so he pays at the same place he pays the ones for them to resell."
			2- Adherence to the healthy living rules/recommendations	2- "... even to cut her nails, we have to take her to the hospital because if she is injured it's going to be painful, because for diabetic people, when they have the injury it does not heal quickly, especially because he saw the mother of one of his friends have this problem and they end up amputated the foot and that's why she says no, to cut my nails, you have to take me to the hospital."
			3- Have an alarm/reminder system that help patient to be adherent to the therapeutic plan	3- "I asked my children to set up an alarm for me for the time I need to take the medication. When I hear the ring, I know this is the time to take my medicine. That's how I do it in the morning, I get up I have my coffee, I take my medicine. At noon, I take my medicine but, in the evening, I have to hear the ring so that I do not forget the time."
			4- Be able to buy all the necessary products and services for the treatment	4- "Just like I showed you, I have the strips for blood sugar, I have the needed devices, I have the needles to inject because if you don't have that, you can't do it at home and for the blood pressure if you don't have the device, what are you going to do? ... I have the devices for blood pressure, I tell you that's what you see in my notebook there ... I paid for my devices, I didn't pay them all at the same time, I paid one after I paid the other one."
		Meaningfulness (5)	1- Feel that healing is possible	1- "… I really believe in the healing process … Me I saw a great improvement, as I came last week Thursday for my result. I went to apply to really see how it works, and I realized that its mostly better, the thing drops down from 2.41 to 1.15 (Blood Sugar)."
			2- Find reasons to fight for recovery and stay healthy	2- "... She is quite positive as mom. She wants to live for herself, for her children, her grandchildren and her great-grandchildren. I think that's what gives her strength ... She is a very positive mom even sometimes when we find that she is tired and must rest, she answers no no no you want me to give up? I have to fight ... she would love to stay with her children and see her grandsons grow up. And she herself knows that she is needed

				and that's why I think she's taking these drugs.”
			3- Fear of dying	3- “She's a mom who's too afraid of death, so she doesn't joke with her medication, no she doesn't even joke a little, she knows that at this time she has to take her medications she has to eat this she has to follow her diet...”
			4- Trust the doctor	4- “... for her the Doctor is like God on the hearth to the point that, all the Doctor ask to do, she does it, even better. For example, they were asked not to eat salty foods, so she only eats foods that have no salt that does not have a cube (spices). For example, yams, eggplants, so that's the only thing she eats at home are vegetables without oil, she consumes it like that, and she is very comfortable.”
			5- Ignoring stigma	5- "She has no worries, she has no problem, she says to herself: if in the village there are thousands of people living with even HIV/AIDS, and we cannot compare hypertension to HIV, so for her if those people who have HIV live and are OK with the situation, why not her. It is both incurable diseases but we cannot compare the incomparable. It is like that; there are other people who are in a worse situation, she is really OK with her disease and she handles it like that."
	GRR (8)	Internal resources (3)	1- Being committed to change and keep healthy lifestyles (Diet, exercise, etc.)	1- “I tell you that in the morning I made carrots’ salad, with diabetics’ sauce [bought] in the local market and I eat as breakfast and not every day. Sometimes if there is remaining food, I take it and eat for breakfast. I also cook the banana with the carrots with the green beans and fish and I do not fry it, I put in like that, I put the peanut oil or olive oil. I make sure I eat enough vegetable or fruits every day. The sport, I always do, I do sports with my friends ... I get up and I go at 6:30 to do sport ... at least 3 times a week to maintain balance.”
			2- Culture of certain regions naturally promotes healthy lifestyle habits	2- “You know that we are people from the northwest region, we eat a lot of vegetables with...” "Because we are BETI people, we also eat too many vegetables and it is also what doctor recommends here. I eat vegetables without salt, I cannot go a week without eating vegetables I drink water they say you have to drink a lot of water.”
			3- Being a positive person	3- She's positive as mom, she's a very positive mom... so she fights a lot to heal from her disease and always expect the good, for that she’s positive and I think she could advise others.”
		External resources (5)	1- Have close family and community support	1- “…neighbours are supportive enough to help her take care of herself... we do care about her, we love her very much,

				sometimes she forgets to take her medication, her nearest neighbour reminds her, recommends her vegetables and encourage her to eat.”
			2- Have a small family (few people under the same roof)	2- I'm lucky that in Cameroon now I only have my wife and a nephew at home because my own children are already old, they are 35-40 years old and live in Europe. So [we are] only tree people at home make it easy to eat what you want, what is recommended. So, I always ask myself if I had my whole family here, 07 children, how was it be possible for me to have a special meal every day?”
			3- Have a dietician	3- “… it's because I didn't follow the recommendation well, I went to the nutrition centre for help, and as I was followed by a dietician the weight dropped constantly"
			4- Have a financial assistance to pay drugs/products/services	4- “They are my children, because whatever the cost of the tests or the drugs is, they pay.”
			5- Have her/his own field	5- “…it helps to have our own field, because in the rainy season we make a lot of reserves for when dry season comes. Me, for example, I have a field with very good vegetables for our health, the “Nkems” (local name of green leaves) and we pick to vary our meals.”
	Adherence and health outcomes (1)		Positive changes in health status following the recommend healthy lifestyles /therapeutic plan.	“… she had a lot of improvement, because before she was overweight but now with the diet to which she is subjected, she lost a lot of weight in less than 2 months, she lost almost 8 kg and that's what is encouraging her so so much.”
	Belief (1)		Believing in God (having hope that everything will be fine and being grateful)	… And I think that since I came here, I thank God, I really felt good following the advices and the training of mother Mary, people will give glory to God about my illness in this hospital…the new nurse I met there just cooled me down and explained to me slowly in such a way that all my fears disappeared…I can be grateful, so we will not go deep into it like that. So, I thank God; I thank all the doctors who are in this hospital.”
	Satisfaction (1)		Good physician–patient relationship	“… I'm fine with the doctor, when I'm with him, he gives me advice and recommendations, he supports me a lot, so the doctors are good here. We are very lucky here as patients... he advises us very well.”
